# An Intelligent Diagnostic System for Thyroid-Associated Ophthalmopathy Based on Facial Images

**DOI:** 10.3389/fmed.2022.920716

**Published:** 2022-06-10

**Authors:** Xiao Huang, Lie Ju, Jian Li, Linfeng He, Fei Tong, Siyu Liu, Pan Li, Yun Zhang, Xin Wang, Zhiwen Yang, Jianhao Xiong, Lin Wang, Xin Zhao, Wanji He, Yelin Huang, Zongyuan Ge, Xuan Yao, Weihua Yang, Ruili Wei

**Affiliations:** ^1^Department of Ophthalmology, Changzheng Hospital, Naval Medical University, Shanghai, China; ^2^Airdoc LLC, Beijing, China; ^3^Faculty of Engineering, Monash University, Clayton, VIC, Australia; ^4^Department of Ophthalmology, Naval Medical Center of the People’s Liberation Army, Naval Medical University, Shanghai, China; ^5^The Laboratory of Artificial Intelligence and Bigdata in Ophthalmology, The Affiliated Eye Hospital of Nanjing Medical University, Nanjing, China

**Keywords:** artificial intelligence, diagnosis, facial images, machine learning, medical data analysis, thyroid-associated ophthalmopathy

## Abstract

**Background:**

Thyroid-associated ophthalmopathy (TAO) is one of the most common orbital diseases that seriously threatens visual function and significantly affects patients’ appearances, rendering them unable to work. This study established an intelligent diagnostic system for TAO based on facial images.

**Methods:**

Patient images and data were obtained from medical records of patients with TAO who visited Shanghai Changzheng Hospital from 2013 to 2018. Eyelid retraction, ocular dyskinesia, conjunctival congestion, and other signs were noted on the images. Patients were classified according to the types, stages, and grades of TAO based on the diagnostic criteria. The diagnostic system consisted of multiple task-specific models.

**Results:**

The intelligent diagnostic system accurately diagnosed TAO in three stages. The built-in models pre-processed the facial images and diagnosed multiple TAO signs, with average areas under the receiver operating characteristic curves exceeding 0.85 (F1 score >0.80).

**Conclusion:**

The intelligent diagnostic system introduced in this study accurately identified several common signs of TAO.

## Introduction

Thyroid-associated ophthalmopathy (TAO) is a common orbital disease (1). Several quality-of-life surveys have shown that the visual function, mental health, and social function of most patients with moderate-to-severe TAO are severely impeded (2–4). Although its clinical manifestations are complex and variable, there are clear diagnostic criteria (5) and management guidelines (6) for this disease. Experienced ophthalmologists are able to rapidly diagnose the type, stage, and grade of TAO and develop a treatment plan to prevent disease progression based on simple interrogation, visual examinations, and basic eye examinations, such as exophthalmometry. However, few ophthalmologists in developing countries have experience diagnosing and treating TAO. In addition, TAO is a complicated disease that can easily be misdiagnosed and mistreated, especially in its early stages.

According to the TAO diagnostic criteria (5), exophthalmos is an important diagnostic sign of TAO. Therefore, the use of facial images can significantly reduce the time required for patient visits and referrals. However, manual diagnosis of TAO *via* facial images is time-consuming and inaccurate (7–13). Approximately 26% of patients receive a final diagnosis after more than 12 months, and many patients with TAO do not undergo treatment at specialized centers or undergo delayed treatment in the late stages of the disease, resulting in an unfavorable disease course (14).

In recent years, the application of artificial intelligence (AI), including deep learning technology, into the field of medical imaging has significantly improved the diagnostic accuracy and efficiency of several diseases, including eye diseases. However, there are no reports regarding the use of computer-aided AI tools for the diagnosis of TAO based on facial images.

A novel AI-based system that incorporates deep learning and machine learning techniques to detect signs of TAO based on facial images is presented in this study.

## Materials and Methods

### Data Collection

The study was conducted in accordance with the principles of the Declaration of Helsinki and was approved by the Medical Ethics Committee of Naval Medical University. Written informed consent for the publication of this study was obtained from all patients.

**FIGURE 1:**
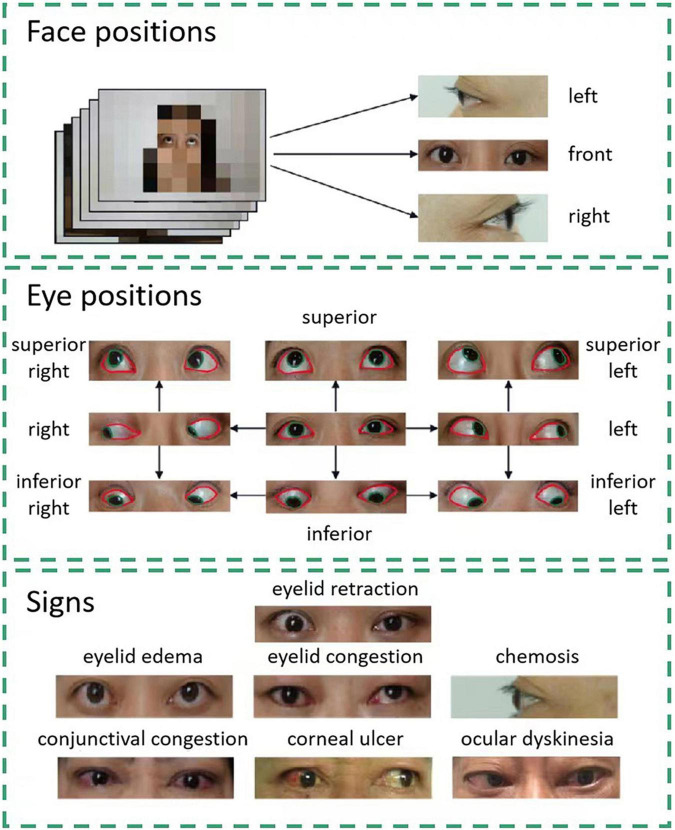
Facial data collection and sign annotation. Images of three face positions and nine eye positions are included in the study. Each eye position image is labeled pixel-wise. The areas of the cornea and sclera are denoted by green and red lines, respectively. Using these images, the system can detect seven signs of thyroid-associated ophthalmopathy.

The data of consecutive patients with TAO treated at Shanghai Changzheng Hospital from January 2013 to January 2018 were included in this study. All facial images were captured using a Sony ILCE-7M2 camera (SONY China Co., Ltd.) [lighting: NG CN-576 (Guangdong Nanguang Film & Television Equipment Co. Ltd.)] during routine examinations. The patient diagnoses were extracted from medical records. No patient had comorbidities that would affect their facial expressions or images. Photographs of the front, right, and left of each patient’s face were obtained (Figure 1). Nine eye positions were photographed from the front: superior right, superior, superior left, right, front, left, inferior right, inferior, and inferior left. Seven common signs of TAO were identified in the photographs: eyelid retraction, eyelid congestion, eyelid edema, conjunctival congestion, chemosis, corneal ulcer, and ocular dyskinesia.

The contours of the cornea and sclera were manually drawn on each image using the open-source interactive software tool LabelMe (15). The annotated areas were then mapped using one-hot encoding in the annotation maps (Figure 1).

Only horizontal and vertical flips were used for data augmentation. As the eye position changes with data augmentation, the corresponding eye position label was also changed when the flipped images were used.

The test population comprised 20% of the total patient population in this study. The remaining 80% of the patient population served as the training set, including 10% that was used for internal cross-validation to determine the best diagnostic model.

### Diagnostic Modules

**FIGURE 2:**
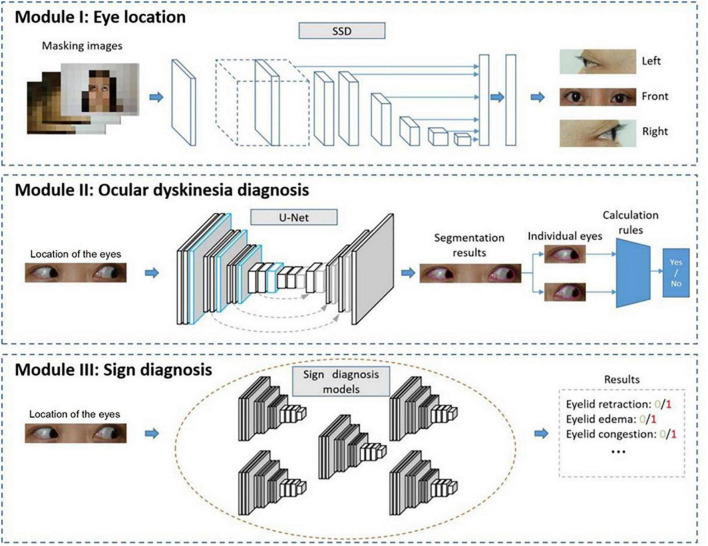
Automated diagnostic system framework. The system framework includes Module I to detect the location of the eyes, Module II to diagnose ocular dyskinesia, and Module III to detect signs of thyroid-associated ophthalmopathy.

The diagnostic methods used in this study included modules based on eye location (Module I), ocular dyskinesia (Module II), and other signs (Module III), as shown in Figure 2.

In Module I, an image of the entire face of the patient was input into a trained detection network that analyzed the location of the eyes. The image was cropped, retaining only the eye area.

**FIGURE 3:**
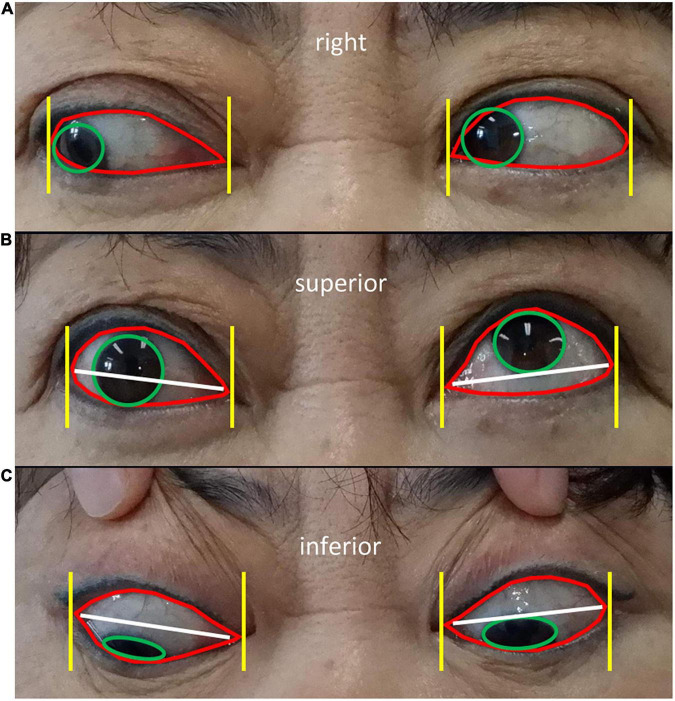
Diagnostic rules of eye movement disorders **(A)** Diagnostic rules of eye movement disorders. Frontal images of left/righ eye rotation show the cornea (green circle in **A**) deviating from the vertex of the canthus (yellow line in **A**), indicating impaired eye movement (left eye in **A**). When the cornea is tangent to or intersects the vertex of the canthus, no eye movement disorders are present (right eye in **A**). Frontal images of superior/inferior eye movement show the cornea (green circle in **B,C**) intersecting the line between the inner and outer canthus (white line in **B,C**), representing impaired eye movement (right eye in **B**). When the cornea is tangent to or separated from the line between the inner and outer canthus, no eye movement disorders are present (left eye in **B** and both eyes in **C**).

Module II aimed to diagnose ocular dyskinesia based on segmentation of the cornea and palpebral fissures in the eye area images obtained in Module I and specific calculation rules (16). Impaired eye movement was based on left/right eye rotation when the cornea deviated vertically from the vertex of the canthus in the frontal photographs (Figure 3A). When the cornea was tangent to or intersected the line at the vertex of the canthus, no eye movement disorder was noted. In frontal images of superior/inferior eye movement, impaired eye movement was identified when the cornea intersected with the line between the inner and outer canthus (Figure 3B). When the cornea was tangent to or separated from the line between the inner and outer canthus, no eye movement disorder was noted (Figures 3B,C).

In Module III, multiple classification networks were trained to diagnose the signs of TAO using the eye images obtained in the first module, such as eyelid retraction, eyelid edema, eyelid congestion, conjunctival congestion, corneal ulcer, and chemosis.

### Deep Learning Networks

Preprocessing is required to remove irrelevant information from facial images and retain only the eye area. A single-shot multi-box detector (SSD) (17) is a simple, effective framework for object detection that is easy to train and straightforward to integrate into systems that require a detection component. The ResNet-50 (18) detection component was trained to detect multiple signs of TAO. One network was used to detect each sign. A semantic segmentation network (U-Net) (19) was trained to identify eye movement disorders in the areas of the cornea and sclera to improve diagnostic accuracy. The experimental environment was built using Ubuntu version 18.04.4 LTS 64-bit with GPU 1080Ti and 11 GB memory. The implementation of deep neural networks was based on PyTorch version 1.6.0 (20).

### Implementation

The captured images were resized to 512 × 512 pixels prior to their use in the SSD (Module I). The cropped eye area images used in U-Net and ResNet were 224 × 224 pixels. The pixel values of each image were normalized from (0, 255) to (0, 1) prior to the training. To expand the available training data and prevent the model from overfitting, the images were augmented using horizontal and vertical flips, translation (−30, 30), and scaling (0.9, 1.1). The stochastic gradient descent optimizer (21) was used for backpropagation to minimize the objective function (cross-entropy loss) in Module I. The learning rate ranged from 1 × 10^–3^ to 1 × 10^–5^ with the division by 10 on epoch 10 and epoch 20. Thirty epochs were trained. The Adam optimizer (22) and a cosine-shaped learning rate ranging from 1 × 10^–3^ to 1 × 10^–6^ were used in Modules II and III. Fifty epochs were trained for Module II and 100 epochs were trained for Module III.

### Evaluation Metrics

Individual models were trained to identify specific signs, and the outcome of the model was based on a yes/no binary classification task. The area under the receiver operating curve (AUROC) quantified the capability of each model to conduct the binary classification, with 0.5 indicating a random chance and 1.0 indicating a perfect model of the validated data (23). The sensitivity and specificity were calculated to evaluate the performance of the diagnostic model.

The accuracy of the eye locations and corneal and scleral segmentation were important for Modules I and II. Accurate eye locations help filter irrelevant information, such as the patient’s face and background. Intersection-over-union (IoU; the Jaccard index) was used to evaluate the detection and segmentation models. IoU, one of the most commonly used metrics in object detection and semantic segmentation tasks, was calculated as:


(1) $$\frac{\mathrm{Area}\,\mathrm{of}\,\mathrm{Overlap}\,\left({\text{A}}_{1},A_{2}\right)}{\mathrm{Area}\,\mathrm{of}\,\mathrm{Union}\,\left({\text{A}}_{1},A_{2}\right)}$$

where A1 is the ground-truth area, and A2 is the prediction of the model.

## Results

### Patient Characteristics

A total of 21,840 images from 1,560 patients (3,120 eyes) were used in this study (Table 1). Eye movement disorders were identified in 77.50% of patients, conjunctival congestion in 62.63%, chemosis in 69.49%, and corneal ulcers in 7.44% (Table 2).

**TABLE 1:** Patient image types.

Types	Number (%)	Sign	Number (%)
Anteroposterior view	1,560 (7.14%)	Eyelid retraction	459 (29.42%)
		Eyelid edema	819 (52.50%)
		Eyelid congestion	241 (15.49%)
		Conjunctival congestion	907 (58.14%)
		Corneal ulcer	93 (5.96%)
Nine eye positions views	14,040 (64.28%)	Eye movement disorders	9,672 (68.89%)
90° lateral view	3,120 (14.29%)	Chemosis	1,237 (39.65%)

**TABLE 2:** Patient characteristics.

Variables		Number (%)
Age (years)	10–19	17 (1.09%)
	20–29	289 (18.53%)
	30–39	384 (24.62%)
	40–49	365 (23.40%)
	50–59	309 (19.81%)
	60–69	159 (10.19%)
	70–79	35 (2.24%)
	80–89	2 (0.13%)
Sex	Male	563 (36.09%)
	Female	997 (63.91%)
Eye pain	Yes	1,278 (81.92%)
	No	282 (18.08%)
Visual loss	Yes	334 (21.41%)
	No	1,226 (78.59%)
Diplopia	No	896 (57.44%)
	Vertical	59 (3.78%)
	Horizontal	12 (0.77%)
	Mixed	593 (38.01%)
Eyelid retraction	Yes/no	479 (30.71%)/1,081 (69.29%)
Eyelid congestion	Yes/no	236 (15.13%)/1,324 (84.87%)
Eyelid edema	Yes/no	870 (55.77%)/690 (44.23%)
Lid lag	Yes/no	388 (24.87%)/1,172 (75.13%)
Lacrimal caruncle edema	Yes/no	24 (1.54%)/1,536 (98.46%)
Eye movement disorders	Yes/no	1,209 (77.50%)/351 (22.50%)
Conjunctival congestion	Yes/no	977 (62.63%)/583 (37.37%)
Chemosis	Yes/no	1,084 (69.49%)/476 (30.51%)
Corneal ulcer	Yes/no	116 (7.44%)/1,444 (92.56%)
Type of TAO	Hyperreflexive	1,324 (84.87%)
	Normal	193 (12.37%)
	Hyporeflexive	43 (2.76%)
Stage of TAO	Active	487 (31.22%)
	Quiescent	1,073 (68.78%)
Grade of TAO	Mild	89 (5.71%)
	Moderately severe	1,290 (82.69%)
	Very severe	181 (11.60%)

*TAO, thyroid-associated ophthalmopathy.*

### Modules I and II

Module I had an accuracy of 0.98 using an IoU threshold of >0.5 (Table 3). Module II had accuracies of 0.93 for corneal segmentation and 0.87 for scleral segmentation.

**TABLE 3:** Accuracy of Modules I and II.

	Accuracy (IoU)
Eye location	0.98 (0.52)
Cornea segmentation	0.93 (0.89)
Sclera segmentation	0.87 (0.89)

*IoU, intersection-over-union.*

### Module III

**FIGURE 4:**
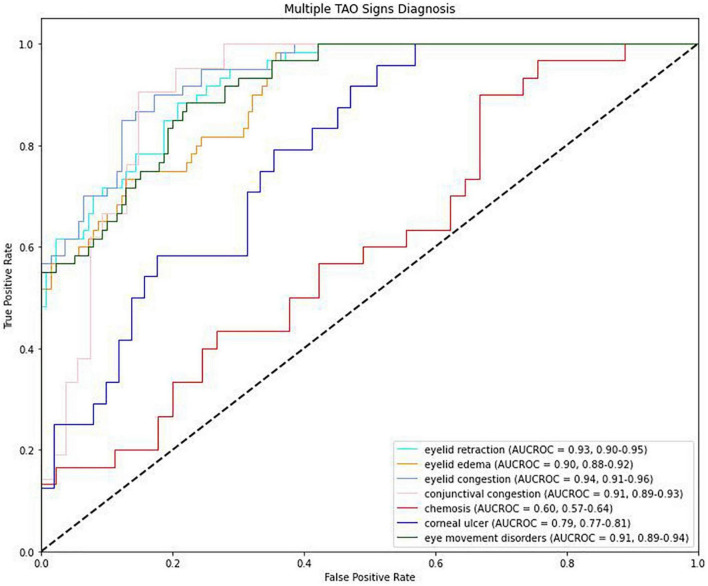
Receiver operating characteristic (ROC) curves for the detection of signs of thyroid-associated ophthalmopathy.

The AUROC for the detection of eyelid edema was 0.90 [95% confidence interval (CI): 0.88–0.93], while that for the detection of chemosis was 0.60 (95% CI: 0.53–0.68) (Table 4 and Figure 4). The AUROC for conjunctival congestion was 0.91 (95% CI: 0.86–0.96) and for eye movement disorders was 0.93 (95% CI: 0.89–0.96). Of the signs present in less than 30% of patients, eyelid congestion had an AUROC of 0.95 (95% CI: 0.90–0.98), eyelid retraction had an AUROC of 0.93 (95% CI: 0.89–0.96), and corneal ulcer had an AUROC of 0.79 (95% CI: 0.76–0.82). The mean AUROC of the seven signs of TAO was 0.85, with a mean sensitivity of 0.80, mean specificity of 0.79, and mean F1 score of 0.80.

**TABLE 4:** Diagnostic performances.

	AUROC (95% CI)	Sensitivity (95% CI)	Specificity (95% CI)	F1 score
Eyelid retraction	0.93 (0.90–0.95)	0.87 (0.83–0.91)	0.88 (0.85–0.91)	0.87
Eyelid edema	0.90 (0.88–0.92)	0.79 (0.75–0.83)	0.86 (0.83–0.89)	0.82
Eyelid congestion	0.94 (0.91–0.96)	0.89 (0.85–0.93)	0.90 (0.87–0.93)	0.89
Conjunctival congestion	0.91 (0.89–0.93)	0.83 (0.79–0.87)	0.85 (0.82–0.88)	0.84
Chemosis	0.60 (0.57–0.64)	0.61 (0.57–0.65)	0.55 (0.52–0.58)	0.58
Corneal ulcer	0.79 (0.77–0.81)	0.77 (0.73–0.81)	0.73 (0.70–0.76)	0.75
Eye movement disorders	0.91 (0.89–0.94)	0.85 (0.81–0.89)	0.79 (0.76–0.82)	0.82
Mean	0.85	0.80	0.79	0.80

*AUROC, area under the receiver operating characteristic curve; CI, confidence interval.*

The use of the ResNet-101 network backbone achieved an AUROC of 0.91 (95% CI: 0.89–0.93) (Table 5). The use of the InceptionV3 network backbone achieved an AUROC of 0.89 (95% CI: 0.87–0.92).

**TABLE 5:** Performance of different backbones.

Backbone	AUROC (95% CI)	Training time	Parameters
ResNet-50	0.91 (0.89–0.93)	×1.0	25.6 M
ResNet-101	0.92 (0.90–0.94)	×1.5	44.5 M
InceptionV3	0.89 (0.87–0.92)	×1.8	27.2 M

*AUROC, area under the receiver operating characteristic curve; CI, confidence interval.*

## Discussion

Thyroid-associated ophthalmopathy is typically accompanied by Graves’ disease and is often missed or misdiagnosed, especially in the early stage.

In the automated diagnostic system presented in this study, Module I located the patients’ eyes and filtered irrelevant information, resulting in images of the eye area that were able to be used in Module II. The use of a binary classification model for diagnosing eye movement disorders was challenging in Module II. Therefore, the calculation rules for the manual diagnosis of eye movement disorders (16) were followed, and a semantic segmentation network for the location of the cornea and sclera were leveraged, providing auxiliary information that allowed for the automated diagnosis of eye movement disorders with high precision. In Module III, specific-task models for the automatic identification of several signs of TAO were created. With the exception of the detection of chemosis and corneal ulcers, the AUROCs for the detection of signs of TAO were >0.9. Chemosis could not be detected on facial images; higher-definition, side-view images of the eye are needed for multi-model fusion to detect chemosis. In addition, corneal ulcers are rare in patients with TAO. The more common signs of TAO, including eyelid retraction, were easily detected using Module III. These results suggest that automated screening of patient images for the most common signs of TAO is a feasible diagnosis method.

Although diagnostic results mainly relied on the outputs of Module II and Module III, the preprocessing conducted in Module I to locate the eyes was essential. The original images captured by cameras were high-resolution, and most deep learning models require an input size less than 1,000 pixels due to the limitation of GPU memories. However, resizing the original image may have masked the signs of TAO as these are mainly located in the eye areas. Module I successfully removed irrelevant information and retained the part of the image containing the eyes. This diagnostic model focused on the critical features of the images, resulting in a multi-stage framework that has the potential to automatically detect signs of diseases based on patient images.

Automated diagnostic systems should be precise and timely. This can be achieved *via* the use of a lightweight but effective model. The Faster R-CNN network (24) has been described for object detection, while the InceptionV3 network (25) is used for classification, and the Mask R-CNN network (26) is used for segmentation. Module I in this study completed a simple task of eye location using the one-stage detection network SSD. The SSD network achieved similarly results as the two-stage networks and had a faster inference speed. Residual networks with deeper layers, such as ResNet-101, did not result in more accurate findings than ResNet-50, although more GPU memories were required. Therefore, the U-Net network was adapted for the segmentation task in Module II. Prior to selecting which model to adapt in a diagnosis system, the designers of the system should consider multiple factors including inference speed in lieu of creating powerful models with massive parameters.

The automated diagnostic system described in this study has several advantages over manual diagnosis. High-speed and accurate results for the detection of several signs of TAO are generated using facial images. The automated diagnostic system presented in this study can be adapted to several platforms, including mobile devices and cloud services. This system may provide automated diagnostic services for patients with TAO or be used for population screening in the future.

This study has some limitations. First, the system has a low diagnostic accuracy for signs of TAO that require auxiliary modalities to aid evaluation, such as chemosis. Second, although the modules were trained using a large-scale dataset, the system may be affected by the imaging environment, resulting in a decline in diagnostic accuracy. The combination of facial images and other data, such as the patient’s chief complaint, should be the focus of future studies.

## Conclusion

The deep learning-based automatic system for the detection of signs of TAO including eyelid retraction, eyelid edema, eyelid congestion, conjunctival congestion, and eye movement disorders based on facial images presented in this study is a cost-effective, accurate method for the auxiliary diagnosis of TAO, especially for ophthalmologists or general practitioners with limited experience in TAO diagnosis.

## Data Availability Statement

The original contributions presented in this study are included in the article/supplementary material, further inquiries can be directed to the corresponding authors.

## Ethics Statement

The studies involving human participants were reviewed and approved by the Medical Ethics Committee of Naval Medical University. The patients/participants provided their written informed consent to participate in this study.

## Author Contributions

XH designed the research study. LJ performed the research and analyzed the data. XW, ZY, JX, LW, and XZ provided help and advice on methodology. XH, JL, LH, FT, SL, and YZ assisted with validation. XH and LJ wrote the manuscript. All authors contributed to editorial changes and read and approved the final manuscript.

## Conflict of Interest

LJ, XW, ZY, JX, LW, XZ, WH, YH, ZG, and XY were employed by Airdoc LLC. The remaining authors declare that the research was conducted in the absence of any commercial or financial relationships that could be construed as a potential conflict of interest.

## Publisher’s Note

All claims expressed in this article are solely those of the authors and do not necessarily represent those of their affiliated organizations, or those of the publisher, the editors and the reviewers. Any product that may be evaluated in this article, or claim that may be made by its manufacturer, is not guaranteed or endorsed by the publisher.
